# Hepatitis C virus core protein impairs metabolic disorder of liver cell via HOTAIR-Sirt1 signalling

**DOI:** 10.1042/BSR20160088

**Published:** 2016-05-20

**Authors:** Zhi-qin Li, Xin-yu Gu, Jin-xing Hu, Yu Ping, Hua Li, Jing-ya Yan, Juan Li, Ran Sun, Zu-jing Yu, Yi Zhang

**Affiliations:** * Department of Infectious Disease, The First Affiliated Hospital of Zhengzhou University, Zhengzhou 450052, Henan, China; † Biotherapy Center, The First Affiliated Hospital of Zhengzhou University, Zhengzhou 450052, Henan, China; ‡ Department of Oncology, the First Affiliated Hospital of Zhengzhou University, Zhengzhou 450052, Henan, China; § School of Life Sciences, Zhengzhou University, Zhengzhou 450052, Henan, China; ║ Key Laboratory of Clinical-Medicine, the First Affiliated Hospital of Zhengzhou University, Zhengzhou 450052, Henan, China

**Keywords:** HCV core protein, HepG2, HOTAIR, metabolism

## Abstract

It has been suggested that Hepatitis C virus (HCV) core protein is associated with metabolic disorders of liver cell. However, the precise mechanism is still unclear. The aim of the present study was to explore the impact of HCV core protein on hepatocyte metabolism by HepG2 and the possible involvement of long non-coding (lnc) RNAs in this process. The effect of HCV core protein on lncRNAs expression was examined with quantitative RT-PCR (qRT-PCR). Manipulation of HVC core protein and lncRNA HOTAIR was to evaluate the role of interaction between them on cell metabolism-related gene expression and cellular metabolism. The potential downstream Sirt1 signal was examined by western blotting and qRT-PCR. Our data suggested that suppression of HOTAIR abrogates HCV core protein-induced reduction in Sirt1 and differential expression of glucose- and lipid-metabolism-related genes. Also it benefits for metabolic homoeostasis of hepatocyte indicated by restoration of cellular reactive oxygen species (ROS) level and NAD/NADH ratio. By manipulation of HOTAIR, we concluded that HOTAIR negatively regulates Sirt1 expression through affecting its promotor methylation. Moreover, overexpression of Sirt1 reverses pcDNA-HOTAIR-induced glucose- and lipid-metabolism-related gene expression. Our study suggests that HCV core protein causes dysfunction of glucose and lipid metabolism in liver cells through HOTAIR-Sirt1 signalling pathway.

## INTRODUCTION

Chronic hepatic infection with hepatitis C virus (HCV) is one of the most common chronic liver diseases and affects 1% to more than 3% residents in different regions of the world [1]. Persistent viral infections tend to induce dysfunction of liver and are responsible for the occurrence of cirrhosis and hepatocellular carcinoma [2,3]. The initially pathological basics for these progresses largely focus on extensive glucose, lipid and cholesterol metabolic abnormalities of hepatocyte in response to chronic HCV infection [4,5]. Several studies have shown that HCV is able to modulate the expression level of the liver metabolism-related protein. HCV core protein-induced serine phosphorylation of insulin receptor substrate-1; therefore, stimulated insulin resistance and caused decreased glucose uptake. This phenomenon was demonstrated by a HCV core protein transgenic mouse model experiment, in which suggested that HCV core protein contributes to the development of insulin resistance through suppressing activation of insulin receptor substrate [6]. As for lipid metabolism, HCV core protein can inhibit both microsomal triacylglycerol (TG) transfer protein activity and secretion of very low-density lipoprotein (VLDL) [7]. However, to data, the clear mechanism underlying this regulatory action has not been defined yet.

Evidence suggests that pathogenesis of metabolic disorders in HCV infection depends on oxidative stress of normal hepatocyte and induce cellular adaptive enzyme-catalysed reaction [8]. Silent information regulator 1 (Sirt1) is such a NAD^+^-dependent histone deacetylase that modulate of cellular metabolism under normal physiology or pathological conditions [9]. Modifications in NAD^+^ levels are likely to be the most important regulators of Sirt1 activity. Moreover, the increase in NADH (a reduced state of NAD^+^) functions as an indicator for metabolic imbalance.

Recently, long non-coding RNAs (lncRNAs), recognized as a new class of transcripts, are universally discovered in the mammal genome and function as the critical regulators of the epigenome [10]. As with microRNAs, lncRNAs are initially used for predicting liver cancer prognosis and regulating pathogenesis of various liver diseases. Also, expression profiles of lncRNA in HCV associated with secondary liver disease are increasingly discovered [11]. In the present study, we reported liver disease related-lncRNAs profile in HCV core protein-transfected HepG2 cells and identified the significantly up-regulation of HOTAIR. Therefore, we further explore the involvement of HOTAIR in HCV core protein-induced dysfunction of hepatocellular metabolism and its potential downstream signalling.

## MATERIALS AND METHODS

### Cell culture

Human hepatocellular carcinoma HepG2 cells were obtained from A.T.C.C. and cultured in Dulbecco's modified Eagle's medium (DMEM) (Life Technologies) supplemented with 10% fetal bovine serum (FBS), 100 U/ml penicillin, 100 mg/ml streptomycin and 5.5 mM D-glucose. All cells were incubated at 37°C in a humidified atmosphere of 5% CO_2_. Cells were subcultured every 3 days for maintaining good growth characteristics.

### Quantitative real time PCR

Quantitative real time PCR (qRT-PCR) was performed to determine expression of lncRNAs HOTAIR, H19, MALAT1, CMPK2, Lethe and BST2HOTAIR, and mRNA expression of Sirt1, PEPCK (phosphoenolpyruvate carboxy kinase), G6P, GLUT2 (glucose transporter 2), SREBP 1C (sterol regulatory element binding protein), FAS (fatty acid synthesis), ACC (acetyl-coenzyme A carboxylase), PPARα (peroxisome proliferator activating receptor), HMGS (HMG-CoA synthase) and CPT1A (carnitine palmitoyl transferase-1). For initial analysis, total RNAs were isolated using TRIzol reagent (Invitrogen) according to instruction's protocols and equal quality of them were applied into reaction of reverse transcription basing on Quanti Tect Reverse Transcription system (Qiagen). The generated cDNA product was put into real time PCR procedures on the basis of a SYBR Premix Ex Taq (Takara) in presence of SYBR green chemistry in an ABI PRISM 7000 (Applied Biosystems). Expression of GAPDH was used as internal control and standardized object gene expression. 5′-GGCGGATGCAAGTTAATAAAAC-3′ (forward) and 5′-TACGCCTGAGTGTTCACGAG-3′ (reverse). Primers for all other genes were referred to [12]. The relative genes expression analysis was performed using SDS 3.1 software (Applied Biosystems).

### Methylation-specific PCR (MS-PCR)

Methylation-specific PCR (MS-PCR) was performed to evaluate the methylation status of Sirt1. Firstly, genomic DNA from the cultured cells was isolated using a Universal Genomic DNA Extraction Kit Ver3.0 (Takara Bio). The generated DNA of each sample was experienced bisulfite modification process as follows: degeneration in 3 mol/L NaOH for 15 min at 37°C; sulfonation of cytosines in 3.6 mol/L sodium bisulfite and 1 mmol/L hydroquinone (Sigma–Aldrich) for 16 h at 55°C. Following, modified DNA samples were desalted using a DNA clean-up system (Promega Corporation). After treatment, Sirt1 was amplified using Takara's Ex Taq™ DNA Polymerase kit basing on two primers of Sirt1 which cover almost the entire CpG rich region of the proximal Sirt1 promoter in Biomedical Instrumentation Center (USUHS). Relative level of MS-PCR products were analysed by a 2% agarose gel and normalized to non-methylation 16S RNA.

### Western blotting

Cells were harvested and washed with PBS for subsequent analysis of protein expression. Initially, protein samples from all experimental protocols were obtained by cell lysis in RIPA lysis buffer in presence of proteinase inhibitor. After abandon of cell debris by centrifugation, protein samples were quantified and equal quality of them were separated by sodium dodecyl sulfate/7.5% polyacrylamide gel electrophoresis (SDS/PAGE). Resolved proteins were then transferred on to PVDF membrane (Millipore). The membrane loaded protein was blocked with 5% nonfat dry milk for 2 h and subsequent one after another incubated with the primary antibody against Sirt1 and core protein (Santa Cruz) overnight at 4°C and a secondary antibody coupled to horseradish peroxidase for 2 h at room temperature. The protein bands were visualized using BeyoECL Plu (Beyotime Biotechnology) in Bio-RAP. Relative expression of Sirt1 was normalized to internal β-actin.

### Reactive oxygen species detection

Cellular reactive oxygen species assay kit (Abcam) was used to detect reactive oxygen species (ROS) production, which is recognized as a marker for hepatocellular oxidative stress. In brief, cells were plated on to glass coverslips and were loaded with chloromethyl 2′,7′-dichlorodihydrofluorescein diacetate (DCFDA) at a final concentration of 20 μM for 30 min at 37°C. The incorporated radioactivity was measured by microplate reader and the signal was read at Ex 485 nm/Em 535 nm. ROS levels were expressed as relative fluorescence intensity and normalized to the control cells.

### NAD+/NADH ratio

For real time monitoring metabolic state of HepG2, the NAD^+^/NADH ratio [13] was measured using the NAD/NADH Assay Kit (Colorimetric) (ab65348; Abcam) in accordance to the manufacturer's instructions. In brief, total NAD^+^ and NADH (NADt) was extracted from the cell pellet by NADH/NAD Extraction Buffer. Before NADH detection, NAD^+^ was decomposed via heating samples to 60°C for 30 min in a water bath. For reaction, samples were mixed with 100 μL of Reaction Mix and 100 μL of Background Reaction Mix, and maintained at room temperature for 5 min to convert NAD^+^ to NADH. The reaction cycle was started when reaction mixture added with 10 μL of NADH developer per well and was carried out at room temperature for 1–4 h. During the reaction, the resorufin fluorescence was continuously read at *A*_450_ nm using a fluorescent plate reader. The NAD/NADH Ratio is calculated as: *NAD/NADH ratio = (NADt - NADH)/NADH*.

### Detection of Sirt1 deacetylases activity

Enzyme activity of Sirt1 activity was measured using a Sirt1 Activity Assay Kit (Fluorimetric) assay kit (ab156065; Abcam) according to the manufacturer's instructions. The reaction mixture that contains Sirt1 assay buffer (5 μL), Fluoro-substrate peptides (100 mM), NAD^+^ (100 mM) and cell extracts was maintained for 30 min at 37°C. The reaction was stopped by addition of Stop Solution. Fluorescence was subsequently measured in microplate reader for 30 min at Ex 350 nm/Em 460 nm. Enzyme activity were expressed as relative fluorescence intensity and normalized to the control cells.

### Transfection

For transfections, approximately 5×10^5^ HepG2 cells were seeded into six-well tissue culture plates for 24 h prior to transfection. For core protein overexpression, HepG2 cells were transfected with 2 mg of pcDNA3.1(-)-core (containing the full-length HCV core gene by insertion of HCV core gene into EcoRI/BamHI site) or pcDNA3.1(-) (worked as control, Invitrogen). For HOTAIR overexpression, HepG2 cells were transfected with pcDNA-HOTAIR that produced by insertion of PCR product of HOTAIR into pcDNA3.1 (Invitrogen) or pcDNA-negative control (worked as control). For knockdown of HOTAIR, HepG2 cells were transfected with si-HOTAIR recombinant plasmid or siRNA control. The transfection procedure was conducted using Lipofectamine™ 2000 (Invitrogen) in accordance with the manufacturer's protocol. After 6 h incubation at 37°C in 5% CO_2_, the cells were washed with 1 PBS and DMEM with serum was added to the cells. After 48 h of transfection, cells were harvest for following analysis.

### Statistical analysis

Results are expressed as the mean±S.D. General statistical was evaluated by SPSS 16.0 software. Significance between groups was determined using Student's *t* test and one-way analysis of variance (ANOVA). A *P* value less than 0.05 was defined as statistical significant.

## RESULTS

### Core protein promotes HOAIR expression in HepG2

**Figure 1:**
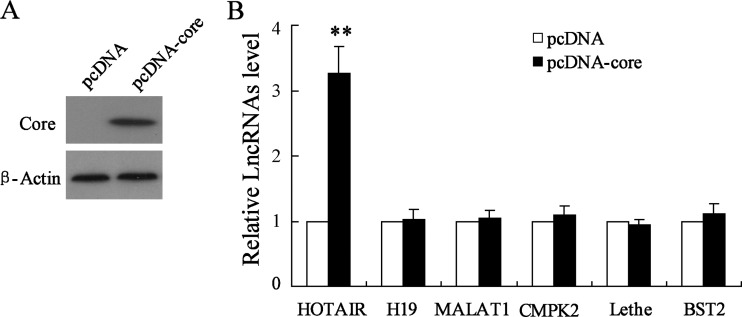
Effect of HCV core protein overexpression on long non-coding RNA expression in HepG2 (**A**) Result from western blot represented core protein expression and (**B**) result from qRT-PCR evaluates relative expression of a series of lncRNA in pcDNA-core transfected HepG2 cells. Data are presented as mean±S.D. **P<0.01 compared with cells transfected with empty pcDNA vector.

We detected ectopic expression of liver disease-specific lncRNAs in HCV core protein overexpression treated HepG2. pcDNA-core transfection led to up-regulation of core protein (Figure 1A). In Figure 1(B), results from qRT-PCR showed that HCV core overexpression contributed to significantly up-regulation of lncRNA HOTAIR; however, it had no effect on relative expression of H19, MALAT1, CMPK2, Lethe and BST2. These data suggested that HOTAIR is a response lncRNA for HCV core protein.

### HOTAIR mediates HCV core protein-induced suppression of Sirt1 expression

**Figure 2:**
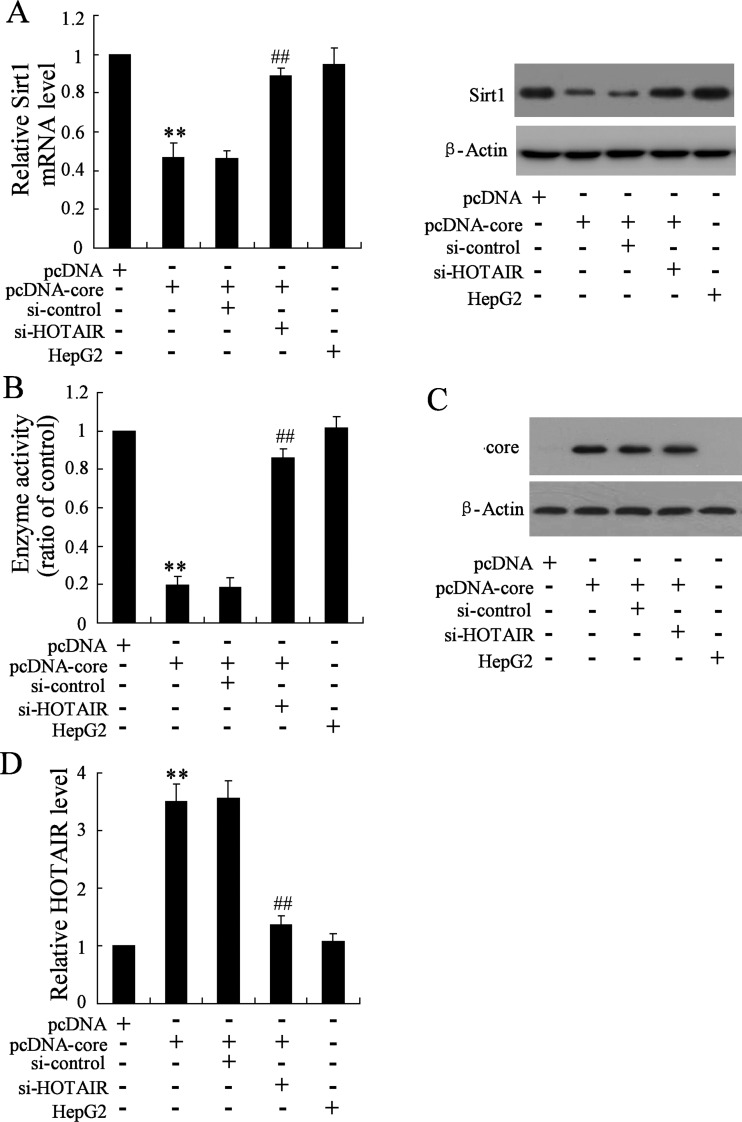
Knockdown of HOTAIR reverses inhibitory effect on Sirt1 expression by core protein (**A**) The expression of Sirt1 mRNA and Sirt1 protein by qRT-PCR and western blot respectively, (**B**) enzyme activity of Sirt1, (**C**) core protein expression and (**D**) HOTAIR expression were determined in HepG2 cells transfected with constructed pcDNA-core, or HepG2 cells co-transfected with pcDNA-core and si-HOTAIR. HepG2 cell with no pcDNA-core and/or si-HOTAIR treatment was as positive control. Data are presented as mean±S.D. **P<0.01 compared with cells transfected with empty pcDNA vector; ^##^*P*<0.01 compared with cells transfected with pcDNA-core and si-control.

To determine the involvement of HOTAIR in harmful role of HCV core protein for liver cell, we co-transfected HepG2 cells with pcDNA-core protein and si-HOTAIR focusing on evaluate the effect of HOTAIR silencing on inhibitory action of Sirt1 expression by core protein. By qRT-PCR and western blotting, we observed that knockdown of HOTAIR significantly elevated Sirt1 mRNA and protein in presence of pcDNA-core protein overexpression (Figure 2A). Meanwhile, enzyme activity of Sirt1 was enhanced (Figure 2B). To exclude the role of HOTAIR on core protein, we next determined the core expression. As shown in Figure 2(C), knockdown of HOTAIR had no effect on core expression. Moreover, comparing with pcDNA-core, si-HOTAIR transfection significantly down-regulated HOTAIR in presence of core protein (Figure 2D). These data suggested the critical role of HOTAIR in core reduced Sirt1 activity.

### Knockdown of HOTAIR improves metabolism status after pcDNA-core protein treatment of HepG2

**Figure 3:**
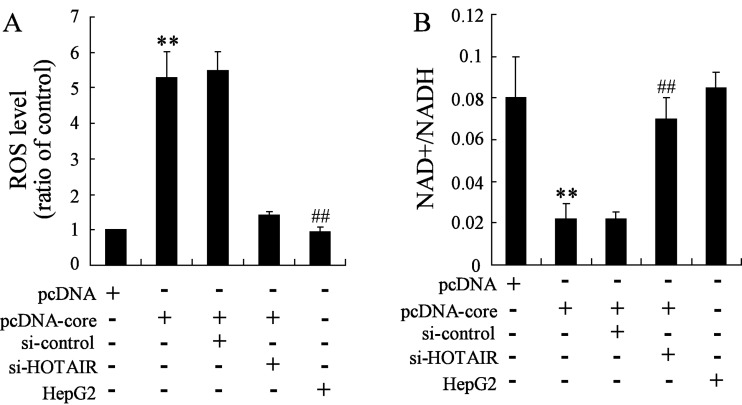
HOTAIR silencing restores metabolism status after pcDNA-core protein treatment of HepG2 (**A**) ROS level and (**B**) NAD^+^/NADH ratio were determined. Data are presented as mean±S.D. HepG2 cell with no pcDNA-core and/or si-HOTAIR treatment was as positive control. **P<0.01 compared with cells transfected with empty pcDNA vector; ^##^*P*<0.01 compared with cells co-transfected with pcDNA-core and si-control.

ROS level and NAD+/NADH ratio were recognized as monitoring indicator for cellular metabolic homoeostasis. Increasing ROS level and NADH represents a status of metabolic imbalance. Therefore, to understand the role of HOTAIR in HCV core protein-induced metabolic disorders, we examined the ROS level and NAD+/NADH ratio in pcDNA-core and si-HOTAIR co-transfected HepG2 cells. The results suggested that ROS is increased in pcDNA-core transfected cell compared with the pcDNA treated cells; however, this elevation is abrogated by knockdown of HOTAIR (Figure 3A). Moreover, inhibitory action on NAD+/NADH by HCV core protein overexpression (compared with pcDNA) is recovered by knockdown of HOTAIR (Figure 3B).

### Differential metabolism-related gene expression in pcDNA-core and si-HOTAIR co-transfected HepG2

**Figure 4:**
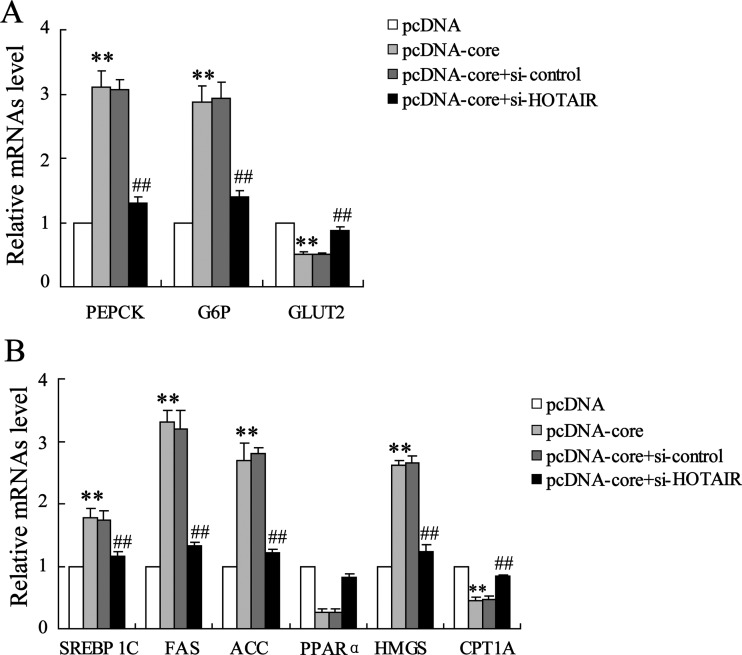
HOTAIR silencing abrogates core protein-induced glucose and lipid metabolism-related gene expression Relative expression of (**A**) glycometabolism-related PEPCK, G6P and GLUT2 mRNA and (**B**) lipid metabolism-related SREBP 1C, FAS, ACC, PPARα, HMGS and CPT1A mRNA were determined in si-HOTAIR and pcDNA-core co-transfected HepG2 cells. Data are presented as mean±S.D. **P<0.01 compared with cells transfected with empty pcDNA vector; ^##^*P*<0.01 compared with cells transfected with pcDNA-core and si-control.

To further confirm the regulatory effect of HOTAIR on hepatocellular metabolism, we next evaluated glucose and lipid metabolism-related gene expression. Results from qRT-PCR analysis suggested that HCV core protein overexpression induced abnormality of glucose metabolism-related gene, that is, up-regulation of PEPCK and G6P and down-regulation of GLUT2 is abrogated by HOTAIR silencing (Figure 4A). Also, aberrant expressions of lipid metabolism-related gene, including SREBP 1C, FAS, ACC, PPARα, HMGS and CPT1A are restored to the original levels (Figure 4B).

### Manipulation of HOTAIR expression negatively regulates Sirt1 expression

**Figure 5:**
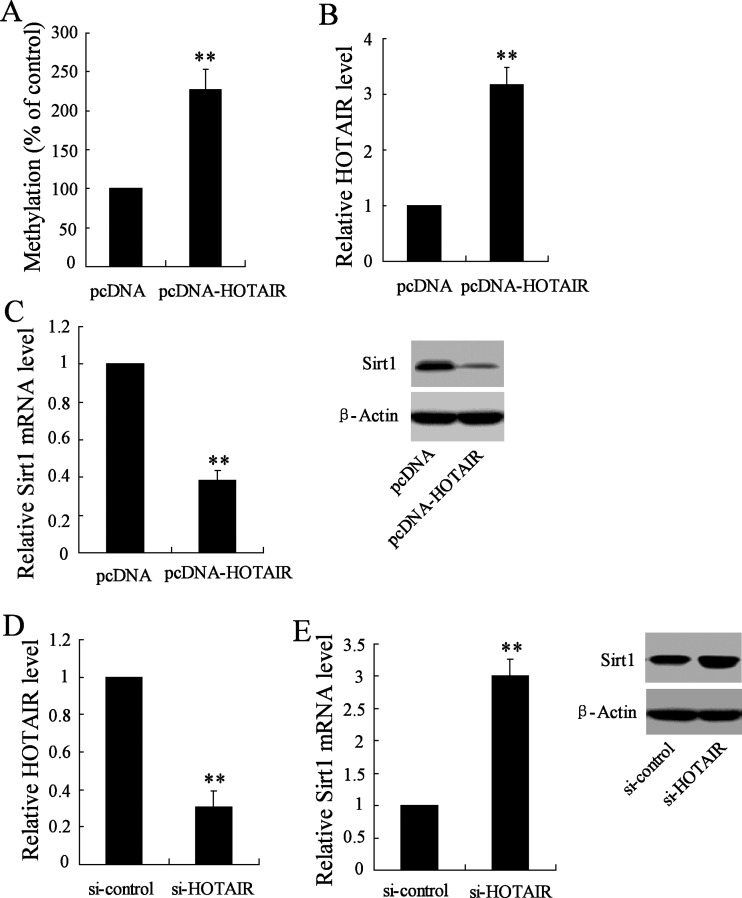
Manipulation of HOTAIR expression affects Sirt1 expression (**A**) MS-PCR was used to evaluate promotor methylation level of Sirt1 in HepG2 cells transfected with pcDNA-HOTAIR. (**B**) Relative expression of HOTAIR was determined in pcDNA-HOTAIR-transfected HepGs cells. (**C**) Analysis of Sirt1 mRNA and protein expression in HepG2 cells transfected with pcDNA-HOTAIR. (**D**) Relative expression of HOTAIR in si-HOTAIR-transfected HepG2 cell. (**E**) Analysis of Sirt1 mRNA and protein expression. **P<0.01 compared with cells transfected with empty pcDNA vector, or si-control.

Considering the aberrant expression of Sirt1 in response to si-HOTAIR treatment, we determined the regulatory role of HOTAIR upon Sirt1 expression through manipulating HOTAIR expression. Our data showed a significant enhancement of methylation of Sirt1 promotor in HepG2 cells in presence of pcDNA-HOTAIR transfection (Figure 5A), which significantly induced HOTAIR expression (Figure 5B). Accordingly, Sirt1 expression in respect of mRNA and protein was suppressed in response to such HOTAIR overexpression (Figure 5C). On the other hand, si-HOTAIR transfection resulted in down-regulation of HOTAIR (Figure 5D), and this knockdown of HOTAIR triggers Sirt1 mRNA and protein expression in comparison with si-control treated cells (Figure 5E).

### Sirt1 mediates HOTAIR overexpression-induced differential metabolism-related gene expression

**Figure 6:**
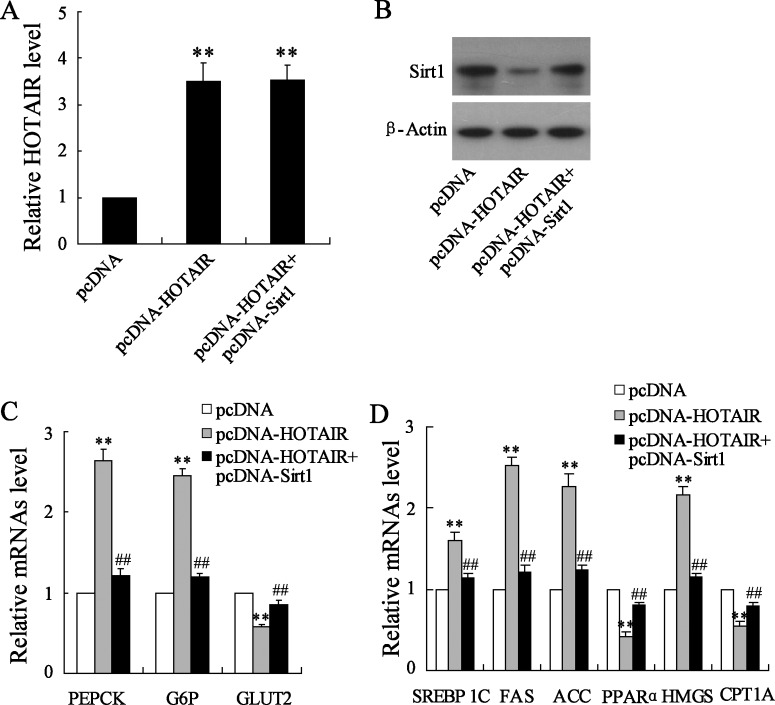
Sirt1 mediates HOTAIR overexpression-induced glucose and lipid metabolism-related gene expression pcDNA-HOTAIR was transfected or co-transfected with pcDNA-Sirt1 into HepG2 cells. (**A**) Relative expression of HOTAIR and (**B**) Sirt1 expression were determined. Relative expressions of (**C**) glycometabolism-related PEPCK, G6P and GLUT2 mRNA and (**D**) lipid metabolism-related SREBP 1C, FAS, ACC, PPARα, HMGS and CPT1A mRNA were determined. Data are presented as mean±S.D. **P<0.01 compared with cells transfected with empty pcDNA vector; ^##^*P*<0.01 compared with cells transfected with pcDNA-core and si-control.

Upon to inhibitory expression of Sirt1 expression by HOTAIR, we thereby explored this interaction in liver cell metabolism by detecting metabolism-related gene expression. Co-transfection of pcDNA-HOTAIR and pcDNA-Sirt1 cannot enhance HOTAIR expression in comparison with pcDNA-HOTAIR alone (Figure 6A); whereas it promotes Sirt1 protein expression (Figure 6B). Expressional variations of glucose metabolism-related PEPCK, G6P and GLUT2 mRNA induced by HOTAIR overexpression are abrogated by pcDNA-Sirt1 treatment (Figure 6C). Similarly, Sirt1 overexpression also restores lipid metabolism-related SREBP 1C, FAS, ACC, PPARα, HMGS and CPT1A mRNA expression in the presence of pcDNA-HOTAIR in HepG2 cells (Figure 6D).

## DISCUSSION

Chronic HCV infection is a significant risk factor for hepatocellular metabolism dysfunction; however, potential signalling connecting this association needs to be further explored. Here, our present study demonstrated the involvement of the fine regulation of Sirt1 protein by lncRNA HOTAIR in cellular metabolism of HepG2 hepatocyte after HCV core protein treatment.

Transcriptional profiling of lncRNAs has identified as an extensive list of differentially expressed regulatory genes [14], including HOTAIR, H19, MALAT1, CMPK2, Lethe and BST2. These genes are increasingly recognized as general transcriptional regulators that are involved in disease development and metabolism [15,16]. However, to data, it remains to be definitely defined whether these developmental gene are merely residual markers of HCV infection. We thus provide new insights on the lncRNA HOTAIR in HCV core protein-stimulated hepatocyte, which showed the up-regulation of HOTAIR in HCV core protein overexpressed HepG2 cells.

Metabolism is a process of comprehensive refection of the catalytic enzyme. Sirt1 is such a nicotinamide adenine dinucleotide (NAD^+^)-dependent histone deacetylase that participant in glucose and lipid metabolism [17]. Sirt1 functions as protective promotor for lipid metabolism. Sirt1 can suppress its target gene SREBP-1c, a key lipogenic activator and regulates hepatic lipid metabolism [18]. Sirt1 activation is demonstrated to be involved in glucose metabolism [19]. Our data suggested that Sirt1 protein and enzyme activity were suppressed by HCV core protein treatment.

Sirt1 activation mediates glucose-induced oxidative stress [20]. Considering the evidence that pathogenesis of metabolic disorders in HCV infection depends on oxidative stress of mitochondrial respiratory chain [21], we thereby detected the oxidative stress status. By results, we observed that HCV core protein induces the alteration of redox state of hepatocytes indicated by increase in ROS level and reduction in NAD^+^/NADH ratio. These finding are consistent with previous studies in which showed that HCV core protein promoted ROS production and declined the NAD+/NADH ratio in core gene transgenic mice as well as in core protein treated HepG2 cells [22].

In consideration of potential correlation between HOTAIR up-regulation and HCV core protein treatment. We simultaneously transfected HepG2 cells with HCV core protein and si-HOTAIR, and observed that HOTAIR silencing partly restores Sirt1 expression and metabolism homoeostasis of hepatic cell in presence of HCV core protein; whereas it exerted no effect on core protein expression. These data suggested that HOTAIR is a responder for core proteins. We also observed that knockdown of HOTAIR reverses HCV core protein-induced aberrant expression of glucose metabolism-related PEPCK, G6P and GLUT2 expression, lipid metabolism-related SREBP 1C, FAS, ACC, PPARα, HMGS and CPT1A expression.

As a basis of gene regulator, lncRNA can mark distinct lysine residues within histone via methylation, leading to inhibition or activation of gene transcription [23]. In this similar trend, using MS-PCR, we observed that HOTAIR elevates methylation level of Sirt1 promotor, with that, causes reduction in transcriptional and translational levels of Sirt1. According, this negatively regulatory action of HOTAIR on Sirt1 expression was further demonstrated by RNA interference analysis, which showed the up-regulation of Sirt1 mRNA and protein in HepG2 cells responding to si-HOTAIR treatment. These data suggested that the pivotal role of HOTAIR in HCV core protein-induced metabolic disorder of hepatocyte and its possible downstream target. We thereby evaluate this regulatory interaction between HOTAIR and Sirt1 in metabolism-related gene expression. By analysis of glucose- and lipid-associated gene mRNA in pcDNA-HOTAIR and pcDNA-Sirt1 co-treated cell, we can confirm the key role of Sirt1 in HOTAIR induced these differential expression of these genes.

In conclusion, our data suggested that, in the *in vitro* model of HepG2 cells overexpressing HCV core protein, up-regulation of HOTAIR may exert its pro-metabolic dysfunction of glucose and lipid in part through the inhibition of Sirt1 protein. Our findings provide a novel recognition of HOTAIR targeting Sirt1 in HCV infected hepatocytes and indicate the potential therapeutic method of HOTAIR expression silencing.

## Abbreviations

HCV: hepatitis C virus
lnc: long non-coding
qRT-PCR: quantitative real time PCR
ROS: reactive oxygen species
TG: triacylglycerol
